# Genetic Anticipation Is Associated with Telomere Shortening in Hereditary Breast Cancer

**DOI:** 10.1371/journal.pgen.1002182

**Published:** 2011-07-28

**Authors:** Beatriz Martinez-Delgado, Kira Yanowsky, Lucia Inglada-Perez, Samuel Domingo, Miguel Urioste, Ana Osorio, Javier Benitez

**Affiliations:** 1 Human Genetics Group, Spanish National Cancer Research Centre (CNIO), Madrid, Spain; 2 Centro de Investigación Biomédica en Red de Enfermedades Raras (CIBERER), Spanish National Cancer Research Centre, Madrid, Spain; 3 Hereditary Endocrine Cancer Group, Spanish National Cancer Research Centre, Madrid, Spain; Stanford University School of Medicine, United States of America

## Abstract

There is increasing evidence suggesting that short telomeres and subsequent genomic instability contribute to malignant transformation. Telomere shortening has been described as a mechanism to explain genetic anticipation in dyskeratosis congenita and Li-Fraumeni syndrome. Since genetic anticipation has been observed in familial breast cancer, we aimed to study telomere length in familial breast cancer patients and hypothesized that genetic defects causing this disease would affect telomere maintenance resulting in shortened telomeres. Here, we first investigated age anticipation in mother-daughter pairs with breast cancer in 623 breast cancer families, classified as *BRCA1*, *BRCA2*, and *BRCAX*. Moreover, we analyzed telomere length in DNA from peripheral blood leukocytes by quantitative PCR in a set of 198 hereditary breast cancer patients, and compared them with 267 control samples and 71 sporadic breast cancer patients. Changes in telomere length in mother-daughter pairs from breast cancer families and controls were also evaluated to address differences through generations. We demonstrated that short telomeres characterize hereditary but not sporadic breast cancer. We have defined a group of BRCAX families with short telomeres, suggesting that telomere maintenance genes might be susceptibility genes for breast cancer. Significantly, we described that progressive telomere shortening is associated with earlier onset of breast cancer in successive generations of affected families. Our results provide evidence that telomere shortening is associated with earlier age of cancer onset in successive generations, suggesting that it might be a mechanism of genetic anticipation in hereditary breast cancer.

## Author Summary

The fact that accelerated telomere shortening accompanies different premature aging syndromes and seems to be associated with increased risk of cancer development prompted us to analyze the role of telomere length in hereditary breast cancer. In this study we found that telomeres of peripheral blood cells from familial breast cancer patients were significantly shorter than those from the control population. Women carrying a mutation in *BRCA1* or *BRCA2* genes, and a subset of BRCAX families, were characterized by short telomeres. We also demonstrated that genetic anticipation, the successive earlier age of onset of cancer, in these families was associated with a decrease of the telomere length in affected daughters compared to their mothers. This is the first study suggesting that telomere shortening may contribute to anticipation in breast cancer families and that analysis of telomere length in hereditary breast cancer may affect the design of surveillance programs for affected families.

## Introduction

Genetic anticipation is the observation of progressively earlier age of onset or an increase of severity of clinical features of a genetic disorder as it is passed on to the next generation. The molecular mechanisms underlying anticipation are largely unknown, but it has been typically associated to trinucleotide repeat expansions in several genetic diseases [1], [2]. In cancer, genetic anticipation has previously been described in several hereditary cancer syndromes, such as hereditary non-polyposis colorectal cancer (HNPCC) [3], [4], familial leukemia [5], Li-Fraumeni Syndrome[6]–[8] and also in familial breast and ovarian cancer [9]–[11].

Telomere shortening has been more recently described as another mechanism of anticipation, being associated with early onset and severity of disease in genetic disorders, such as dyskeratosis congenita [12], [13], a disease characterized by cutaneous abnormalities, bone marrow failure and an increased predisposition to cancer, and in the Li-Fraumeni Syndrome [7], [8]. Families with dyskeratosis congenita have mutations in genes in the telomerase or shelterin complex, causing reduced telomerase activity. Telomeres are nucleoprotein structures that protect the end of chromosomes. Telomeres shorten with each cell cycle and there is increasing evidence suggesting that short telomeres and subsequent genomic instability contribute to malignant transformation.

In this way, data from several case-control studies have indicated that individuals with relatively short mean telomere lengths might have an increased risk for developing cancer [14], [15]. In particular, telomere length and risk of breast cancer have been evaluated in different studies, although disparities among the results did not allow final conclusions [16]–[19].

Inherited predisposition to breast cancer accounts for approximately 5% of all cases and is characterized by an autosomal dominant pattern of inheritance, young age of onset and bilateral breast cancer. Familial breast and ovarian cancer (FBOC) is associated with inherited mutations mainly in two genes, *BRCA1* and *BRCA2*. Women who have inherited mutations in either one of these genes have a high risk of developing breast cancer, ovarian cancer, and several other types of cancer during their lifetimes. However, a large proportion of familial breast cancer is not caused by mutations in *BRCA1* or *BRCA2*. These non-*BRCA1/2* breast cancer families (referred to as BRCAX families) comprise a histopathologically heterogeneous group, further supporting their origin being from other genetic events [20].

Telomere length maintenance is a complex process controlled by a large number of different proteins, and in addition to telomere binding proteins, many other proteins commonly involved in DNA repair are also found at telomeric ends [21]. *BRCA1* and *BRCA2* genes are involved in repair of DNA double strand breaks. Inherited defects in these genes lead to chromosomal instability contributing to malignant cell transformation. Importantly, there is evidence that BRCA1 localized at telomeres and may regulate telomere length and stability [22]–[24]. In addition BRCA2 has been very recently described to be implicated in telomere replication [25].

Based on all these data we hypothesized that telomere shortening may be associated to age anticipation in hereditary breast cancer. In this study, we analyzed telomere length in DNA from peripheral blood leukocytes in familial breast cancer cases and evaluated generational changes in telomere length in mother-daughter pairs with the aim to investigate the role of telomere shortening and its potential implication as a mechanism of age anticipation in familial breast cancer.

## Results

### Anticipation in the age of breast cancer onset in familial breast cancer

**Figure 1:**
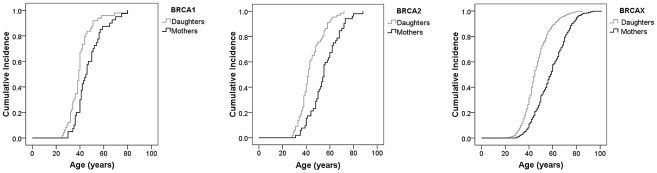
Anticipation effect in the age of breast cancer onset in the familial breast cancer genetic subgroups. Kaplan–Meier curves showing the differences in age of onset of familial breast cancer in mothers and daughters in BRCA1 (left panel), BRCA2 (middle panel), and BRCAX (right panel) families. Log-rank test *p-*values are represented for each group.

The occurrence of age anticipation in mother-daughter pairs with breast cancer was analyzed in 623 FBOC families, classified as BRCA1 (40 families), BRCA2 (52 families), and BRCAX (531 families). The distributions of age at diagnosis for mother and daughters showed a consistent shift to earlier ages in daughters (Figure 1). Evidence for anticipation comparing the age of breast cancer diagnosis in daughters and respective mothers from these families was found after *t*-test in the three genetic groups. Breast cancer was diagnosed at an average of 6.8 years earlier in daughters in the BRCA1 families (p = 0.002) (Table 1). In the BRCA2 and BRCAX groups, a more significant earlier age of diagnosis in daughters was found, being 12.1 years in the BRCA2 (p = 2.9×10^−7^) and 12.3 years in the BRCAX families (p = 1.5×10^−55^). Therefore, significant differences were found in the age of onset between mothers and daughters in the three groups, showing the BRCA2 and BRCAX groups stronger apparent anticipation effect than BRCA1 group.

**Table 1:** Difference in the age of onset of breast cancer between mothers and daughters in hereditary breast cancer.

	Mothers			Daughters						
	n	Mean age (years)	±s.d.	n	Mean age (years)	±s.d.	Mean age difference	±s.d	*t-statistic*	*t-test p value*
***BRCA1***	40	47.6	±11.8	49	40.2	±10.2	6.8	±16.2	3.165	*0.002*
***BRCA2***	52	55.6	±12.7	66	44.1	±10.2	12.1	±14.0	5.196	*2.9×10* ^−*7*^
***BRCAX***	531	58.4	±13.9	643	46.4	±11.1	12.3	±16.7	16.369	*1.5×10* ^−*55*^

Mean age of cancer development in mothers and daughters in the three groups, BRCA1, BRCA2 and BRCAX, of hereditary breast cancer. Statistical significance of the difference between mothers and daughters was estimated by t-tests.

s.d. Standard deviation. Bilateral *t*-test was performed to assess the significance of the differences between mothers and daughters. Values for the *t*-statistic and the *p*-value for the *t*-test are indicated.

### Telomere length of hereditary and sporadic breast cancer cases

**Figure 2:**
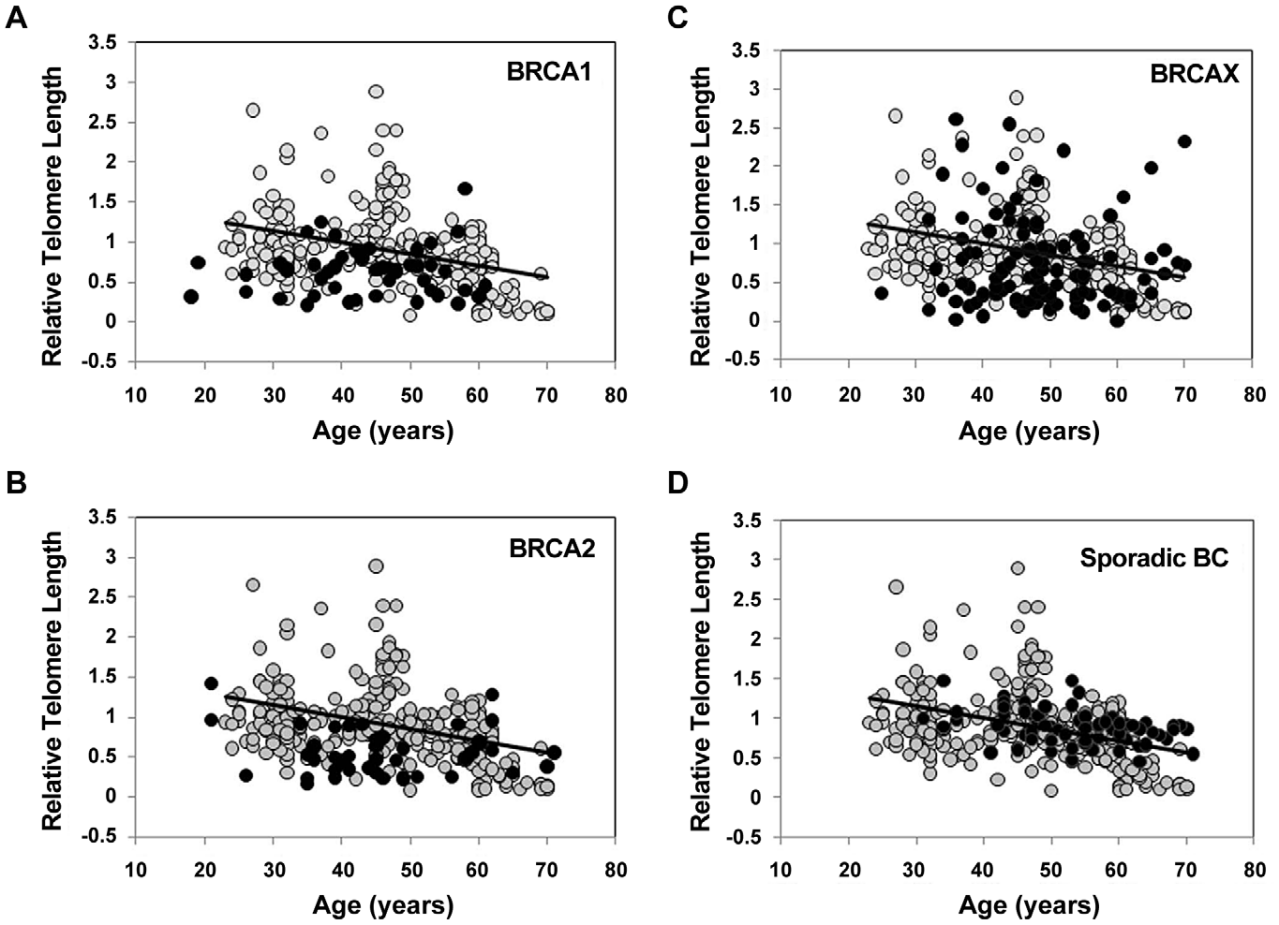
Telomere length distribution in control, familial breast cancer, and sporadic breast cancer cases. Telomere length distribution in peripheral blood leukocytes as a function of age for the control women population (n = 267, grey circles) and for breast cancer cases (black circles). Controls show the expected telomere length erosion with increasing age. Regression line for controls is drawn (y = −0.0146x+1.585, R^2^ = 0.144). Comparison between control telomere length distribution and telomeres from 48 BRCA1 (A), 44 BRCA2 (B), 105 BRCAX (C), and 71 sporadic breast cancer cases (D) is represented.

**Figure 3:**
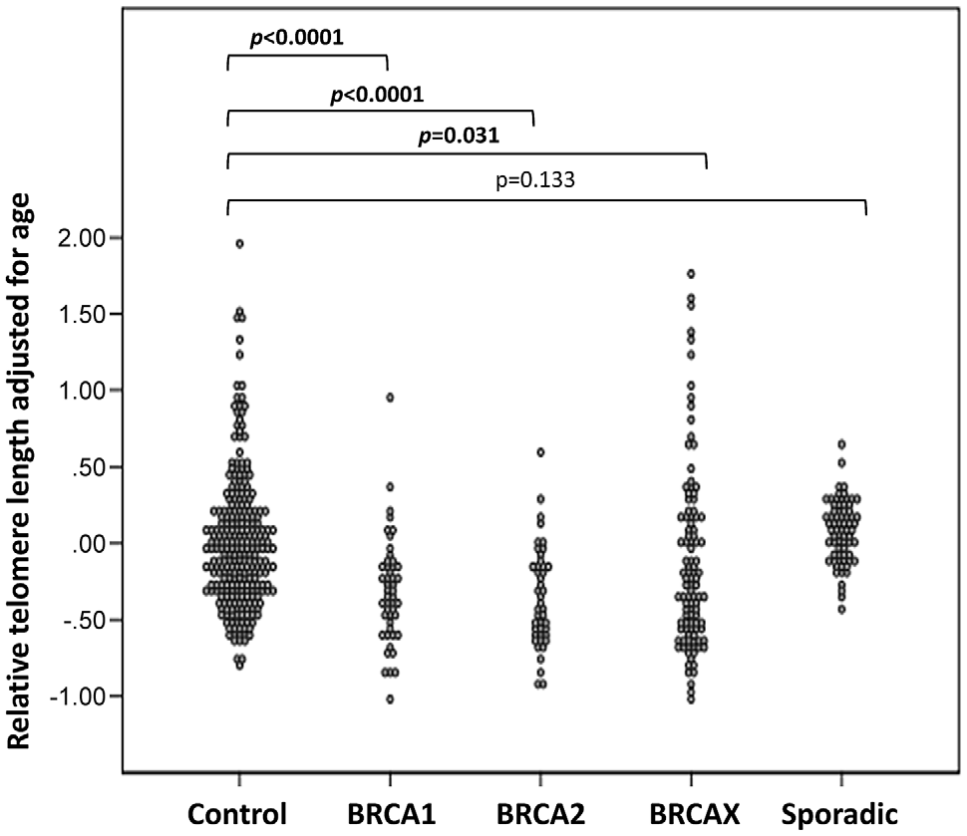
Comparison of telomere length, adjusted for age, in control women and hereditary and sporadic breast cancer. Age-adjusted telomere length (calculated as the difference between the observed and the predicted values; see Materials and Methods section) distribution in peripheral blood leukocytes of control women (n = 267); familial breast cancer groups BRCA1 (n = 48), BRCA2 (n = 45), and BRCAX (n = 105); and sporadic breast cancer (n = 71). Bilateral *t*-tests were performed to compare telomeres of breast cancer groups with the controls and the respective *p*-values are represented. Significantly shorter telomeres were found in all hereditary breast cancer groups whereas no significant differences appeared between controls and sporadic breast cancer cases.

In order to evaluate the hypothesis that telomere shortening may be associated with the earlier onset of the disease, we investigated the mean relative telomere length of blood leukocytes in index cases from FBOC families, either BRCA1 (48 cases), BRCA2 (45 cases), or BRCAX (105 cases), and compared with the relative telomere length of a normal population of healthy women covering an age range between 23 and 70 years (267 samples) (Figure 2). Interestingly, telomere lengths in affected individuals from BRCA1 and BRCA2 families were significantly shorter than those in the control population after adjustment for age using the line of best fit from controls (p<0.0001) (Figure 3). High risk BRCAX families showed a more heterogeneous distribution of telomere length, with cases of both short and long telomeres. Adjusting for age, this group also demonstrated significant differences to the controls (p = 0.031) (Figure 3). We also compared the mean telomere length in blood leukocytes from a group of 71 sporadic breast cancer patients. Importantly, age-adjusted telomere length distribution in these cases did not differ from controls (p = 0.133) (Figure 2 and Figure 3). Therefore, hereditary but not sporadic breast cancer seems to be characterized by short telomeres, primarily in *BRCA1* and *BRCA2* mutation carriers, but also in a subgroup of BRCAX.

### Telomere length in mutation carriers and non-carriers from BRCA1/2 families

**Figure 4:**
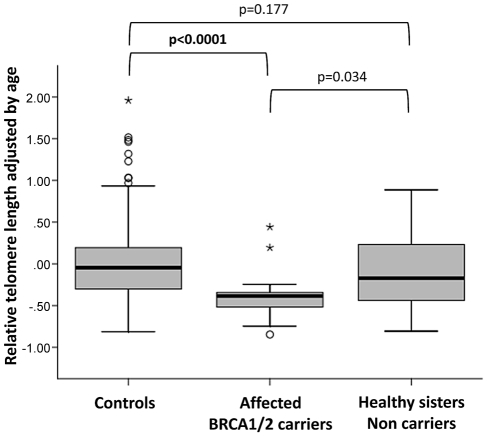
Telomere length in affected BRCA1/2 mutation carriers and corresponding non-carrier sisters. Comparison of age-adjusted telomere length between *BRCA1/2* mutation carriers (n = 19) and healthy non-carrier sisters (n = 22) from 19 different families (8 BRCA1, 11 BRCA2) is represented through box-plots showing the median and interquartile distance for each group. Age-adjusted telomere length was calculated for each sample as the difference between the actual and the predicted value using the line of best fit from controls (see Materials and Methods). Atypical (circles) and extreme (asterisks) values are also shown. Mann-Whitney U tests between the groups were performed, and significantly shorter telomeres were found in mutation carriers versus non-carriers. The non-carrier sisters from affected families do not show differences in telomere length with the control women.

Since *BRCA1* or *BRCA2* mutations were found associated with short telomeres, we further explored whether the altered function of these genes would be causing a reduction in telomere length or whether short telomeres were the consequence of other genetic or environmental factors present in these families. We analyzed telomere length of 19 affected women carrying *BRCA1/2* mutations (8 *BRCA1* and 11 *BRCA2*) and 22 sisters from the same families (8 *BRCA1* and 14 *BRCA2*) who did not inherit the mutations (Figure 4). Affected *BRCA1/2* mutation carriers showed not only significantly shorter telomeres than the unrelated normal controls (p<0.0001) but also versus the healthy sisters not carrying the mutation (p = 0.034). Telomeres of these healthy sisters do not significantly differ from the normal control population (p = 0.177) suggesting that short telomeres are not likely the result of a predisposing genetic background or environment, which was shared by mutation carrier and non-carrier sisters, but would rather be a consequence of the mutation in the *BRCA1/2* genes. So, it seems that haploinsufficiency for *BRCA1* or *BRCA2* in heterozygous women contributes to progressive telomere shortening at a somatic and germline level, affecting the age of cancer onset in successive generations. Because in the BRCAX group the causing mutations are unknown, a similar study cannot be performed, but it is possible that at least a subgroup of BRCAX families characterized by shorter telomeres were associated to mutations in other genes with a role in telomere maintenance.

### Stratification of BRCAX families by telomere length

**Figure 5:**
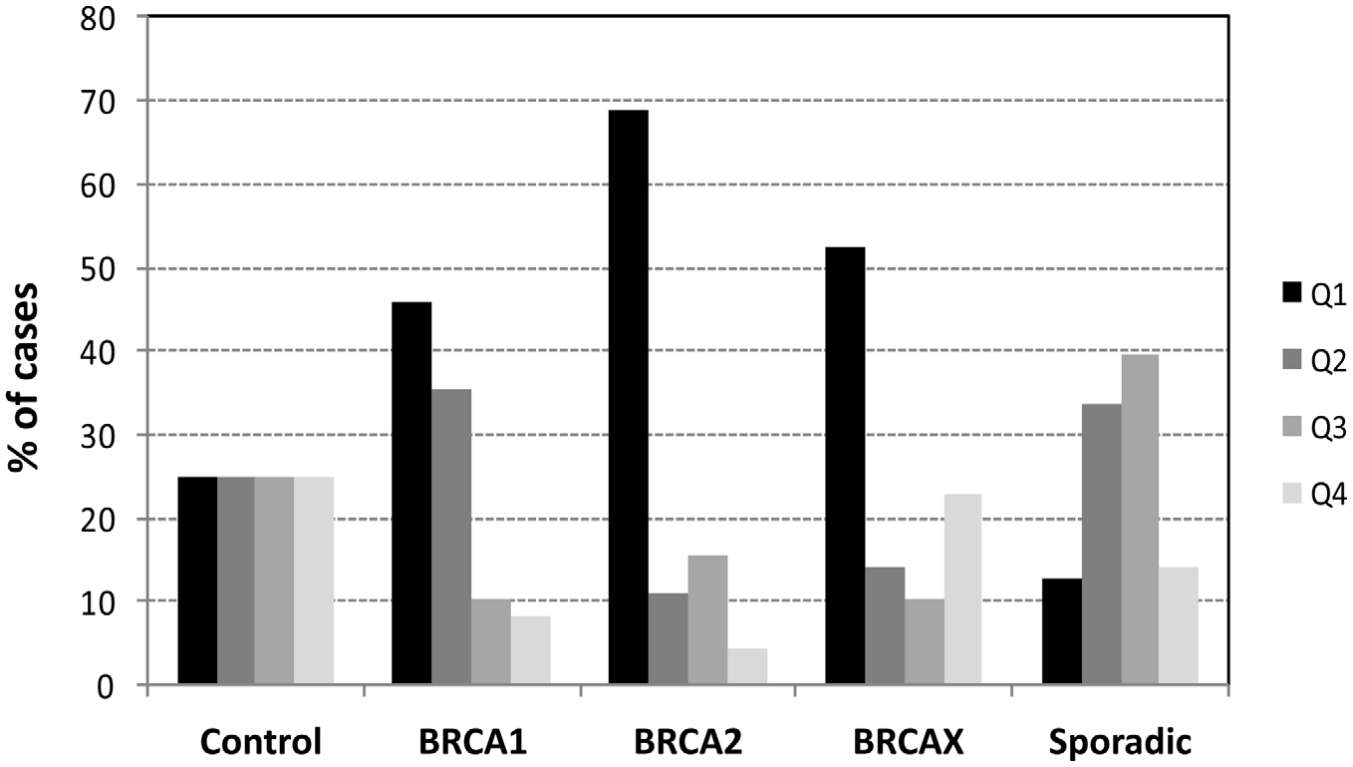
Distribution of hereditary and sporadic breast cancer cases by quartiles of telomere length. First quartile (Q1), characterized by the shortest telomeres, was significantly increased in the three hereditary groups, but decrease in the sporadic breast cancer. For analysis of differences in the proportion of cases in Q1 in breast cancer groups and controls, Chi-square test two-sided was performed.

To better characterize the subgroup of BRCAX families with short telomeres, we divided breast cancer cases into quartiles of telomere length, according to the telomere length distribution in control samples (Figure 5). A significant increase of the first quartile (shortest telomeres) representing 50–70% of the cases in the BRCA1, BRCA2, and BRCAX groups was observed (p = 0.003, p<0.0001, and p<0.0001, respectively). In contrast, the proportion of cases in the first quartile significantly decreased in the sporadic breast cancer group compared to the controls (10% of cases, p = 0.0026). These results suggested that familial breast cancer, BRCA1 and BRCA2, but also a subgroup of BRCAX, was characterized by short telomeres in peripheral blood cells. We further stratified BRCAX families based on the number of generations in which individuals with breast cancer appeared (Table 2). Thus, there were families in which the proband's generation was the first one with affected individuals, and families in which, in addition to the proband's generation, there were one or two additional generations with cancer. Interestingly, those BRCAX families with only one generation affected corresponded almost completely to the fourth quartile characterized by the longest telomeres (Table 2), suggesting that shortening of telomere length might be occurring in successive generations.

**Table 2:** Distribution of BRCAX families in the quartiles of telomere length.

	1 Generation affected		2 Generations affected		3 Generations affected	
Quartiles	*n*	*(%)*	*n*	*(%)*	*n*	*(%)*
**Q1**	0	(0)	26	(50)	23	(53)
**Q2**	0	(0)	12	(23)	10	(23)
**Q3**	1	(11.1)	5	(10)	3	(7)
**Q4**	8	(88.9)	9	(17)	7	(16)
**Total**	9		52		43	

BRCAX families were stratified according to the number of generations with affected breast cancer cases and distributed into the quartiles of telomere length.

### Changes in telomere length in mother-daughter pairs from breast cancer families

**Figure 6:**
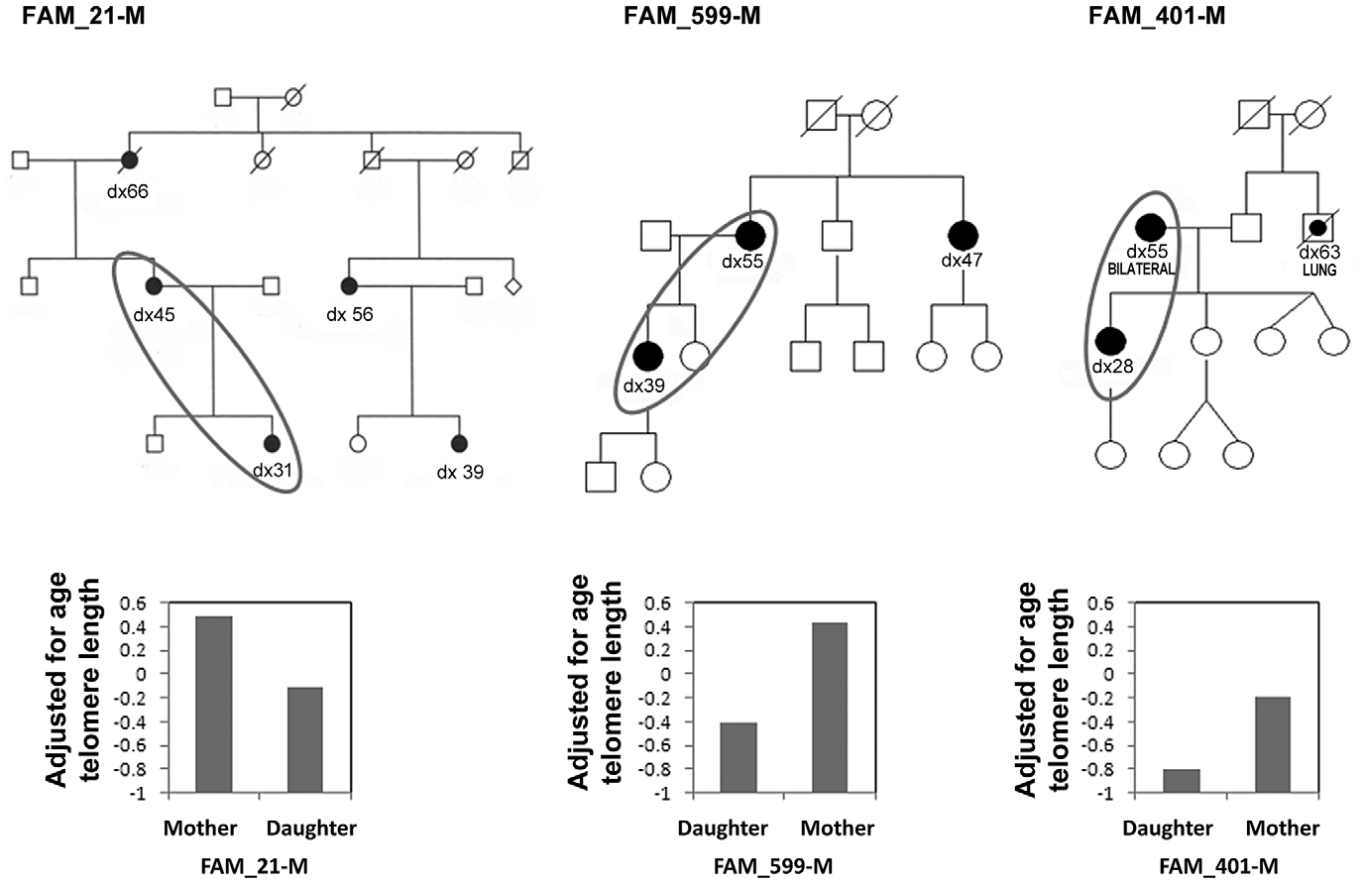
Familial breast cancer pedigrees showing anticipation in the age of onset of breast cancer. Open circles (females) and squares (males) represent normal individuals and black circles represent affected females. Age of breast cancer diagnosis is shown for affected women. Mother-daughter pairs where the telomere length was studied are highlighted and age-adjusted telomere length for each pair is represented in the graphic below the pedigrees.

**Figure 7:**
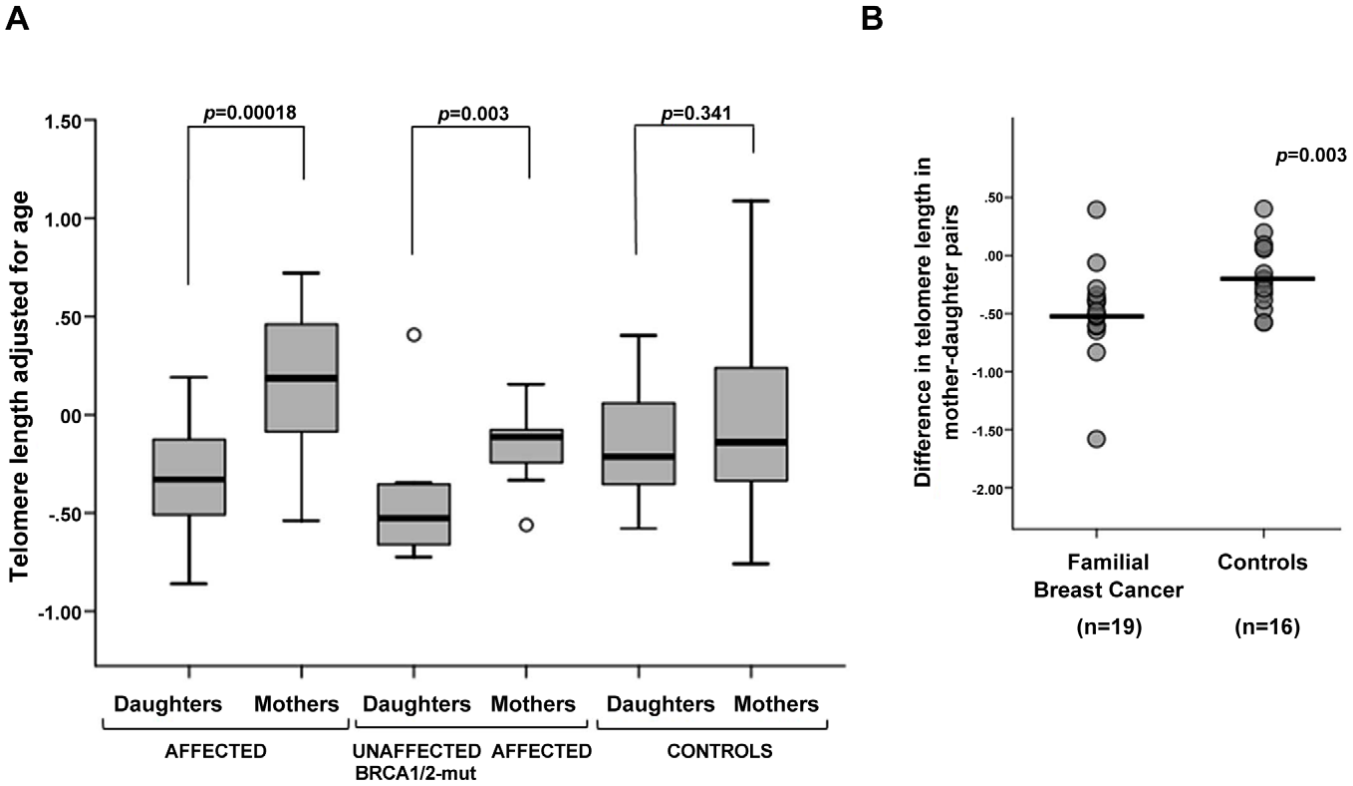
Changes in telomere length between mother-daughter pairs. (A) Box-plots representing telomere length distribution in mothers and daughters affected with breast cancer, affected mothers and daughters unaffected but carrying *BRCA1/2* mutations, and control mothers and daughters. Significant differences were found in mother-daughter pairs from familial breast cancer families with a decrease in the telomere length in both affected and unaffected, mutation-carrying daughters, but not in controls. (B) Generational changes in telomere length in mother-daughter pairs with familial breast cancer (n = 19) compared to control mother-daughter pairs (n = 16). Differences between age-adjusted telomere length in daughters and age-adjusted telomere length of the corresponding mother were obtained for affected and control groups. Median value is drawn as a horizontal line for each group. Telomeres of affected daughters were significantly shorter than those of their mothers, compared to the changes observed in control mother-daughter pairs (Mann-Whitney U, p-value = 0.003).

We next investigated the relation between telomere length and genetic anticipation in breast cancer. We measured changes in telomere length in 19 mother-daughter pairs from FBOC families (3 BRCA1, 1 BRCA2, and 15 BRCAX) who developed breast cancer (Figure 6) (Table 3) and compared them to 16 normal mother-daughter pairs. We additionally analyzed telomeres in 12 pairs of affected mothers from BRCA1/2 families and their respective daughters who were mutation carriers but did not develop cancer to date (Table 4). Telomere length was adjusted for age and we evaluated telomere differences between mothers and daughters in the three groups: both mothers and daughters affected, affected mothers and unaffected mutation-carrying daughters, and mother and daughter controls (Figure 7A). Interestingly, telomere length significantly decreased not only in affected daughters (p = 0.00018) but also in unaffected daughters who were carriers of BRCA1/2 mutations (p = 0.003), while the change between control mothers and daughters was not statistically significant (p = 0.341). This indicates that telomere shortening was associated in these mother-daughter transmissions to the inheritance of a genetic mutation rather than with the disease. Looking at the difference in telomere length of mother-daughter pairs (Figure 7B), significantly larger differences in pairs from affected families were found compared to controls (p = 0.003), with telomeres being shorter in daughters of breast cancer families compared with normal mother-daughter pairs. This decrease in telomere length in successive generations suggests that telomere length could explain age anticipation in familial breast cancer.

**Table 3:** Age-adjusted telomere length and anticipation in the age of diagnosis in mother-daughter pairs.

			Mother			Daughter		
	Fam	Type	Tumor	Age at diagnosis (years)	Telomere (age-adjusted)	Tumor	Age at diagnosis (years)	Telomere (age-adjusted)
								
1	1190-M^a^	BRCA1	BR+OV	44	−0.18229	BR Bi	45	−0.49235
2	1190-M	BRCA1	BR+OV	44	−0.18229	BR Bi	31	−0.52641
3	1195-M	BRCA1	BR Bi	54	0.51041	BR	28	−0.1384
4	0921-M	BRCA2	BR Bi	46	0.02561	BR	36	−0.45342
5	192-M^a^	BRCAX	BR	42	0.72125	BR	42	−0.8601
6	330-M	BRCAX	BR	55	−0.05267	BR	42	−0.56179
7	401-M	BRCAX	BR Bi	55	−0.18586	BR	28	−0.79633
8	1109-M	BRCAX	BR	70	−0.30786	BR	40	−0.37053
9	21-M	BRCAX	BR	45	0.48848	BR	31	−0.11345
10	1528-03-M	BRCAX	BR	52	0.36643	BR	45	−0.14667
11	1885-04-M^a^	BRCAX	BR	50	0.59976	BR	60	0.19095
12	199-M	BRCAX	BR	59	−0.5391	BR+Leu	24	−0.1421
13	599-M	BRCAX	BR	55	0.42882	BR	39	−0.40432
14	621-M	BRCAX	BR	59	0.43238	BR	25	0.0499
15	902-M	BRCAX	BR	51	0.18553	BR+Mel	40	−0.32889
16	934-M	BRCAX	BR	70	0.59847	BR	43	0.07029
17	988-M^a^	BRCAX	BR	35	−0.04272	BR	37	−0.55533
18	1288-M	BRCAX	BR Bi	51	0.28299	BR	33	−0.10973
19	1227-M	BRCAX	BR	74	0.0569	BR	43	−0.28305

Data from the mother-daughter pairs with familial breast cancer showing the age of breast cancer diagnosis and the age-adjusted telomere length.

BR: Breast cancer, BR Bi: Bilateral Breast cancer, OV: Ovarian cancer, Leu: Leukemia, Mel: Melanoma

a Families not showing anticipation in the age of onset.

**Table 4:** Age-adjusted telomere length in mother-daughter pairs with unaffected BRCA1/2 carrier daughters.

			Mother			Daughter		
	Fam	Type	Tumor	Age at diagnosis (years)	Telomere (age-adjusted)	Tumor	Age at interview (years)	Telomere (age-adjusted)
								
1	0901-M	BRCA1	BR	52	0.15442	−	25	−0.71764
2						−	28	0.40677
3	1190-M	BRCA1	BR+OV	44	−0.18229	−	42	−0.36214
4	0951-M	BRCA1	BR+OV	41	−0.33339	−	21	−0.72402
5	1521-M	BRCA1	BR	39	−0.2273	−	27	−0.70288
6	0836-M	BRCA2	BR	41	−0.56158	−	24	−0.34418
7	1145-M	BRCA2	BR	57	−0.11092	−	41	−0.34752
8						−	37	−0.48726
9	0921-M	BRCA2	BR Bi	46	0.02561	−	31	−0.58487
10	1456-M	BRCA2	BR Bi	48	−0.11339	−	26	−0.61877
11	1005-M	BRCA2	BR	69	−0.07681	−	35	−0.56699
12						−	32	−0.40273

Data from the mother-daughter pairs from familial breast cancer families showing the age of breast cancer diagnosis, or age at interview of unaffected daughters, and the age-adjusted telomere length.

BR: Breast cancer, BR Bi: Bilateral Breast cancer, OV: Ovarian cancer.

## Discussion

Telomere dysfunction seems to underlie the development of a range of human genetic, degenerative, aging diseases and cancer [26]. Our results demonstrated that short telomeres in peripheral blood cells were a feature of hereditary breast cancer patients and that telomere shortening frequently occurred with successive generations in these families, suggesting that telomere shortening could be the mechanism to explain the phenomenon of age anticipation in this disease.

Decreasing age of onset in families with hereditary breast cancer has been observed before [9]–[11]. Similar to what we have found, Paterson et al. [10] reported earlier age of diagnosis, of between 6 and 9 years, in successive generations of breast cancer families. Another study performed in a smaller number of BRCA1, BRCA2 and BRCAX families demonstrated that the mean maternal age at diagnosis in the BRCA1 group was significantly lower comparing to the BRCA2 or BRCAX groups, and no significant difference was found in the mean age at diagnosis between mothers and daughters in BRCA1 families [9]. In our study we detected lower anticipation effect in BRCA1 families comparing to BRCA2 or BRCAX. Since BRCA1 mutations predispose to breast cancer at an earlier age, it would be more difficult to have large differences between generations in BRCA1 families. However, there are not definitive conclusions about whether there exists a real anticipation phenomenon. We cannot exclude that there would be alternate explanations for the earlier age of diagnosis in daughters. This observation could be due to ascertainment bias of subjects and lead-time bias, as a result of early detection of cancers by extensive screening or surveillance programs in high risk families [27], [28]. More sophisticated statistical approaches and mechanistic studies are warranted to answer this complicated problem. To date there are studies for [10], [29], [30] and against [28] a real phenomenon of anticipation in breast cancer, but a final conclusion regarding anticipation is still an open matter that is complicated by the fact that there is no molecular mechanism to explain it. Importantly we provide data indicating that telomere shortening is a possible biological explanation for the complex phenomenon of anticipation in breast cancer.

There are several studies trying to find association between short telomeres and risk of breast cancer that have reported contradictory results [16]–[19], [31], [32]. The fact that sporadic breast cancer showed normal telomere length distribution in our study agrees with previous results from case-control studies, focused on sporadic cases, indicating lack of association between telomere lengths in blood leukocytes with risk of breast cancer [31], [32]. Interestingly, one association study reported significant association with breast cancer risk in women under 50 years of age, but no association between telomere length and breast cancer in women 50 years of age or older [17]. Hereditary breast cancer, which we found characterized by short telomeres, typically occurs in women under 50 years. Then, it seems that hereditary breast cancer is associated with short telomeres, especially in *BRCA1* and *BRCA2* mutation carriers, as well as in a subgroup of BRCAX.

It has been reported that telomere attrition may be affected by factors such as smoking, oxidative stress or obesity [33]–[35]. Recently a prospective case-control study in breast cancer suggested that telomere shortening mainly occurs after diagnosis, as an effect of chemotherapy or other aspects of disease progression [16]. However, the observed telomere shortening in unaffected BRCA mutation carrier daughters, as well as shorter telomere length in hereditary versus sporadic cases, although they followed similar therapeutic strategies, indicate that telomere shortening is largely influenced by genetic events. Nevertheless, the results are limited by the absence of data regarding timing of the sample draw, and the application of any chemotherapy. Our results showing that telomeres of affected women from *BRCA1* and *BRCA2* families were significantly shorter than normal controls suggest that these genes might be involved in telomere regulation. Similarly, we can speculate that other genes involved in telomere maintenance could also be susceptibility genes that explain at least part of the BRCAX families with short telomeres.

Telomeres were shorter in affected mutation carriers versus non carriers and, moreover, we found that telomeres shortened in successive generations in affected families comparing with controls, suggesting that this is a plausible mechanism explaining the observed anticipation effect in familial breast cancer. We can speculate that mutations in *BRCA1/2* or other genes induce faster telomere attrition during life time increasing the probability of genomic instability and the risk of developing breast cancer. Breast cancer would develop, first as a consequence of the inherited mutation, as this is the critical risk factor, and then the telomere length could modify the age at which the cancer would appear, as shorter telomeres at birth would reach dramatic genetic instability earlier that longer ones. On the other hand, telomere shortening could be a process mainly associated with the presence of mutations in *BRCA1/2,* or some other genes, regardless of the disease pathophysiology or age of presentation. Interestingly, we found four families in which daughters showed shorter telomeres than their mothers although the age of breast cancer was not anticipated with respect their mothers (Table 4), indicating that other genetic modifiers or environmental factors would be also determining the age of onset of breast cancer.

Telomere length variations as well as different telomere erosion rates were observed within the different lymphocyte subsets [36], [37]. In our study we used unselected peripheral blood leukocytes to estimate telomere lengths and then it could be affected by cell-to-cell variations. Selection of specific lymphocyte subpopulation would provide a more refined knowledge of the role that telomere length could have in cancer risk assessment.

The fact that telomere shortening has been associated with anticipation in other diseases, such as dyskeratosis congenita [13], as well as Li-Fraumeni Syndrome [7], suggest that telomere shortening may be responsible for genetic anticipation in a wide spectrum of genetic diseases.

Our findings indicate that the study of telomere length would be of relevance in the clinical surveillance and design of appropriate screening tests for patients with familial breast cancer.

## Materials and Methods

### Samples

Informed consent was obtained for all patients involved in this study and the research project has the approval of the ethics committee of our institution. Families used in this study were selected from the register of the Familial Cancer Consultation of the CNIO Human Genetics Group. All of them fulfilled the high risk criteria for genetic testing [38]. Index cases had been screened for mutations in *BRCA1* and *BRCA2* by a combination of DHPLC and direct sequencing as previously reported [39]. A total of 623 breast cancer high-risk families (40 *BRCA1*, 66 *BRCA2*, and 531 families without mutations in *BRCA1/2*) which included 758 mother-daughter pairs affected with breast cancer were selected to analyze the anticipation effect in the age of onset. To avoid differences in the age of onset of different types of tumors occurring in these families, i.e. breast, ovary, and other tumors, only mother-daughter pairs who developed breast tumors were used to estimate the anticipation effect.

Telomere length in familial breast cancer cases was analyzed using DNA extracted from peripheral blood leukocytes in index cases from a set of 198 Spanish breast cancer families corresponding to 48 BRCA1, 45 BRCA2, and 105 BRCAX families. In these three groups the distribution of ages at which the telomere were analyzed was for BRCA1 a mean of 43 years (range, 18–61), mean 48 years (range, 21–71) for BRCA2 cases and for BRCAX cases a mean of 48 years (range, 25–70). These samples corresponded to women with familial breast cancer who met the high risk criteria and attended the Spanish National Cancer Centre family cancer clinics between 2002 and 2009. In addition, samples from 8 affected *BRCA1* mutations carriers and sisters not carrying the mutation, and 11 samples from affected *BRCA2* mutations carriers and 14 healthy sisters without mutation from these families were included to study the relation between telomere length and the presence of an inherited mutation under the same genetic background.

DNA samples from 267 control women with a mean age of 46 years (range, 23 to 70 years) were also analyzed to compare normal telomere length distribution with that in breast cancer patients. Controls corresponded to Spanish healthy women without personal or familial antecedents of cancer, recruited at different Hospitals in Spain for different epidemiologic studies [40]. Age distribution of controls was homogeneous enough to demonstrate the expected decline of telomere length with age.

Seventy-one peripheral blood samples from a group of women with sporadic breast cancer at ages ranging from 31 to 61, mean 53 years, were also analyzed. These sporadic cases were consecutive newly diagnosed breast cancer patients, without familial antecedents of breast cancer, recruited between 2006 and 2007 in the different Hospitals in Madrid. These cases were included in previous studies from our group [40], [41].

### Telomere-length quantification

Genomic DNA was automatically extracted from peripheral blood mononuclear cells using the MagNA Pure LC 2.0 System (Roche). Telomere length was measured using a quantitative PCR-based technique previously described [42], [43]. By this method telomere length is calculated as a ratio between telomere repeat copy number (T) and a single-copy gene, *36B4*, copy number (S). Primers used to amplify telomere repeats and the *36B4* gene were described before [43]. DNA samples were amplified in a total reaction volume of 10 µl containing 1x Power SYBR Green PCR Master Mix (Applied Biosystems), 300 nM of primer Tel1, 900 nM of primer Tel2, and 30 ng of DNA. For *36B4* reactions the concentrations of primers were 300 nM of 36B4u and 500 nM of 36B4d. All samples, for both telomere and *36B4* amplifications, were analyzed in triplicate using an ABI 7900HT thermal cycler, in 384-well format. A robot Biomek NXp (Beckman Coulter) was used to load DNA and PCR mixes into the 384 PCR plates. The thermal cycling profile was the same for both assays: 95°C incubation for 10 minutes followed by 35 cycles of 95°C for 15 seconds, 54°C for 2 minutes, and 72°C for 15 seconds.

Each PCR reaction plate included two samples (DNA from the MBA-MD-436 cell line) to be used for inter-run calibration. DNA from the cell line MBA-MD-436 was used to construct a standard serial dilution series (1/4 dilutions starting from 50 ng) for PCR efficiency calculation. We observed that over 100 ng linearity is lost, and therefore samples were diluted to 10 to 30 ng for proper measurements. The amplification efficiencies (*E*) of each PCR were calculated from the slopes of the standard curves according to *Eff* = 10^(−1/slope)^. The efficiency was calculated for each plate, both for the telomere and for the *36B4* signal. PCR data was analyzed using the SDS 2.2.2 program. The threshold value was established in the initial part of the exponential phase of the amplification curves and the crossing of this line with the curve defines the threshold cycle value (*Ct*) for all samples. For each sample the relative concentration of both Telomere (T) and *36B4* (S) was calculated relative to the calibration sample and PCR efficiency to obtain the T/S ratio, as previously described [14], applying the following formula:
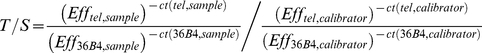



Concordance among triplicates was checked, and the coefficient of variation was obtained for Telomere (average 0.85%, range 0.2–2.8%) and 36B4 (average 0.54%, range 0.12–1.5%). Reproducibility was tested by repeating two samples in all experiments. Good agreement between measurements were found throughout the experiments (*r* = 0.86).

### Statistical analysis

To asses telomere length differences between control samples and hereditary and sporadic breast cancer cases, telomere length measurements were adjusted for age using the line of best fit for controls. Thus, the difference between the actual and the predicted value was calculated for each sample. Differences in age-adjusted telomere lengths were analyzed by bilateral *t*-tests.

The Kolmogorov-Smirnov test was used to evaluate normality in telomere length of *BRCA1/2* mutation carriers and their corresponding non-carrier sisters. As a normal distribution could not be assumed, a Mann-Whitney U test was applied to evaluate distribution differences. A similar analysis was done in order to assess generational differences in telomere length between control mother-daughter pairs and mother-daughter pairs from FBOC families. Statistical calculations were performed using SPSS version 17 (SPSS Inc, Chicago, Illinois). Nominal two-sided P-values less than 0.05 were considered statistically significant.

Due to limitations of previous information regarding telomere length in the population studied, a post hoc power analysis for comparisons of telomere length among the different groups was made. Power analysis for the test between controls and sporadic cases, which showed no significant differences in telomere length, was 63%.
